# Goos–Hänchen shift of inelastically scattered spin-wave beams and cascade nonlinear magnon processes

**DOI:** 10.1038/s41598-025-86879-y

**Published:** 2025-02-14

**Authors:** Krzysztof Sobucki, Igor Lyubchanskii, Maciej Krawczyk, Paweł Gruszecki

**Affiliations:** 1 Institute of Spintronics and Quantum Information, Faculty of Physics, Adam Mickiewicz University, Uniwersytetu Poznańskiego 2, 61-614 Poznań, Poland; 2 in association with Adam Mickiewicz University, Poznan, Poland

**Keywords:** Magnetic properties and materials, Spintronics, Surfaces, interfaces and thin films

## Abstract

We study, using micromagnetic simulations, the inelastic scattering of spin-wave beams on edge-localized spin-wave modes in a thin ferromagnetic film. In the splitting and confluence processes, the new spin-wave beams are generated with frequencies shifted by the edge-mode frequency. We report that inelastically scattered spin-wave beams in both processes not only change their direction of propagation but also undergo lateral shifts along the interface, analogous to the Goos–Hänchen effect known in optics. These shifts of inelastically scattered beams, for a few special cases described in the paper, can be in the range of several wavelengths, which is larger than the Goos–Hänchen shift of elastically reflected beam. Unexpectedly, at selected frequencies, we found a significant increase in the value of the lateral shifts of the scattered spin-wave beams formed in the confluence process. We show that this effect is associated with the cascading nonlinear processes taking place at the edge of the film and involving the primary edge spin wave. Our results make an important contribution to the understanding of the nonlinear nature of spin waves and provide a way to exploit it in signal processing with magnons.

## Introduction

Spin waves (SWs), propagating precessional magnetization disturbances, are a promising candidate for information carriers, especially in the context of their applications in beyond-CMOS^1^ and artificial neural networks^2,3^. One of the SWs advantages is their intrinsic nonlinearity, a key element for their advanced applications such as neuromorphic computing^4,5^. Another advantage is the possibility of the SWs confinement in a small part of a magnetic material allowing for the large miniaturization of the magnonic devices. For instance, SWs may be confined in a nanoscale-wide potential well induced by the static demagnetization field near the layer’s edge^6–9^. Such edge-localized SW modes are called edge SWs or edge modes, and usually, their frequencies are lower compared with the SWs propagating outside of the well. Recently, there has been considerable attention devoted to the research on inelastic scattering of SWs on localized modes, mostly to obtain frequency combs^10–13^, but also for other applications, like sensing^14^ or SW demultiplexing^15^. Several spatially localized modes on which propagating SWs are inelastically scattered have been considered. These include, nonlinear scattering on the skyrmion gyrotropic mode^11,16,17^, azimuthal SWs in vortex^13,18–20^, domain wall mode^12,21,22^, and the SW edge mode^15^. In the last case, the inelastic scattering of an obliquely incident SW beam of frequency *f* at the edge of a Permalloy (Py) thin film on a propagating SW edge mode of frequency $\nu$ results in two primary three-magnon processes, i.e., stimulated splitting process^23–25^ (SSP) and confluence process^23,24^ (CP). CP causes two modes at frequencies *f* and $\nu$ to merge (confluence) into a new SW at frequency $f+\nu$. On the other hand, SSP causes the splitting of the mode at frequency *f* into two modes at frequencies $f-\nu$ and $\nu$ with the assistance of the edge mode at frequency $\nu$, which stimulates this process and increases the intensity of the new waves. It has been shown that SSP could be used to realize the magnon-based transistor^26^, while both SSP and CP could be used for demultiplexing^15^. In the second example, the scattered SW beams created in nonlinear processes propagate under different angles compared to the reflected SW beam and the angles of propagation depend on the edge mode frequency $\nu$. The mechanism of this phenomenon was explained by employing the isofrequency contours analysis and conservation of the tangential component of beam wavevectors^27,28^.

The reflection of the wave beam from the edge of the material is in some cases associated with non-specular behavior well-known as the Goose-Hänchen (GH) effect as first predicted, observed and described in optics^29–32^ and manifests itself as a spatial shift of a totally internally reflected light beam along the interface. The origin of this effect is the phase acquisition of the waves during reflection. In optical experiments, the GH shift ranges from a few nanometers (fractions of light wavelengths) up to a few micrometers^33–36^. In magnetic materials the magneto-optical GH effect was theoretically studied in Refs.^37–41^ and experimentally observed for BK7 prism/Fe/Au^42^ and Ni-based magneto-plasmonic crystals^43^. Since the GH effect is due to the wave nature, the analogous effect has been observed for different types of waves. For instance, the GH effect has been confirmed for plasmons^44–46^, electrons^47^, neutrons^48^, seismic waves^49^ and SWs^50–55^. Theoretical predictions have recently shown that an analogous phenomenon to the GH shift can also be found for inelastically scattered electromagnetic waves during Brillouin scattering on phonons^56^. However, to date, there has been no report on the GH effect of the inelastically scattered SWs.

In this paper, we report the GH shift for the SW beam that is inelastically scattered on the propagating edge mode. We numerically demonstrate this effect in an in-plane magnetized thin Py film for the beams generated in the SSP and CP processes taking place at the very edge of the film. We show that the edge mode frequency and wavevector, as well as the angle of propagation of the incident SW beam, affect the GH shift. Unexpectedly, we found a significant enhancement of the GH shift of the SW beam generated in the CP process at a certain frequency of the edge mode. We show that this effect is related to the cascade of three nonlinear processes involving the edge mode and generated high-frequency SW leaky waves propagating into the film. These results demonstrate new effects in magnonics and provide a basis for unexplored methods of SW beam control in thin ferromagnetic films, which can be exploited for practical applications in magnonic technology for high-frequency and low-energy computing systems.

## Results

**Figure 1:**
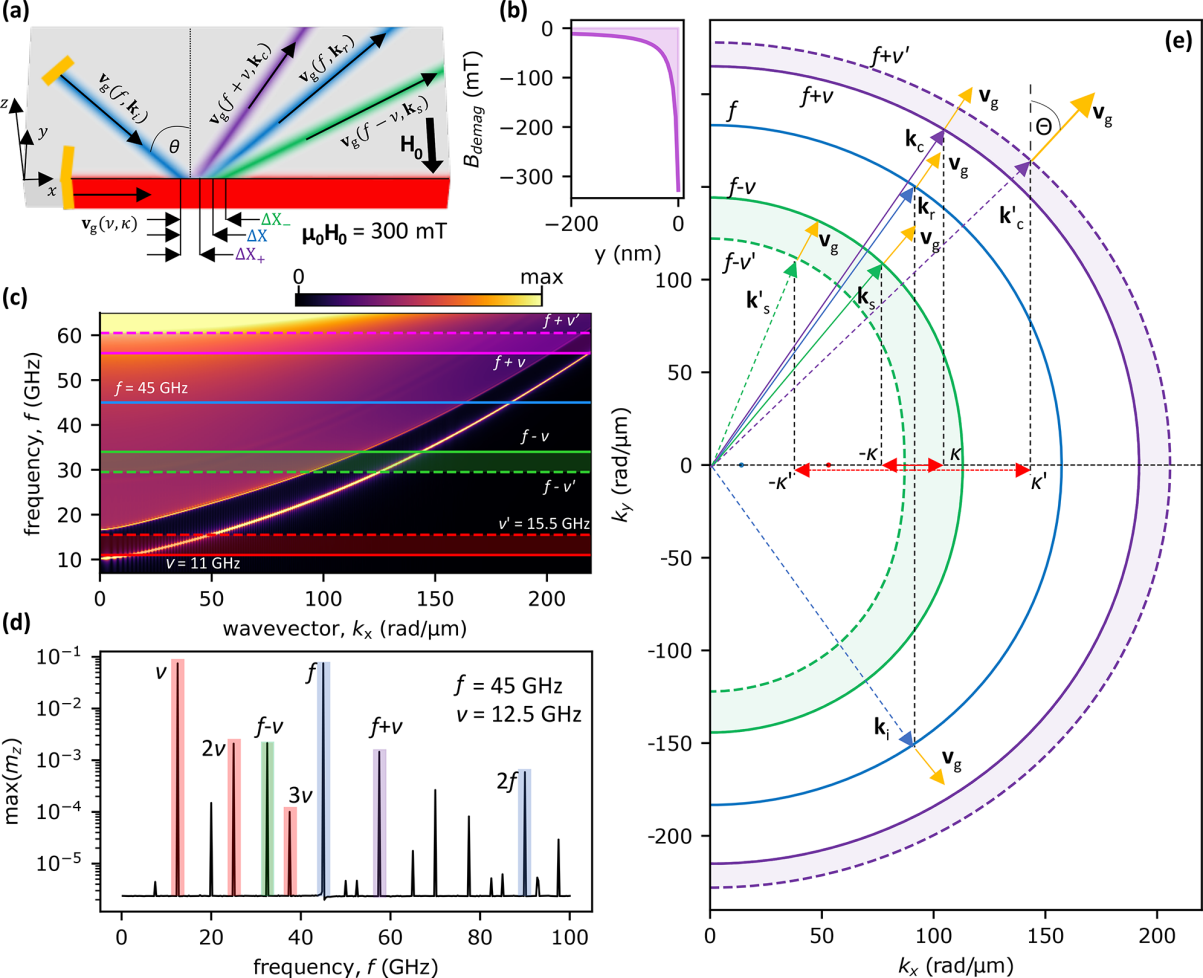
System’s geometry and dispersion relation with isofrequency contours construction. **(a)** Geometry of the investigated system, i.e., Py layer with thickness 10 nm placed in a uniform external magnetic field $\mu _0 H_0=300$ mT along the *y* axis with a schematic representation of SWs propagating at different frequencies. The blue color represents incident and elastically reflected beams. The purple and green colors represent inelastically scattered beams as a result of CP and SSP, respectively. The red color depicts the edge mode. The black arrows denote the directions of the group velocities $\textbf{v}_\textrm{g}$ associated with the respective SWs. The orange lines represent the antennas used to excite the incident SW beam and the edge modes. $\Delta {X}$, $\Delta {X}_{-}$, and $\Delta {X}_{+}$ represent the lateral displacement between the incident beam spot and the beams from reflection, SSP, and CP, respectively. **(b)** Demagnetization field drops at the edge of the system, facilitating the localization of the SW edge mode in the system. **(c)** Numerically calculated dispersion relation in dependence on the tangential to the interface component of the wavevector. The color-shaded areas on the plot indicate investigated ranges of frequencies. **(d)** Spectrum of the SWs for the system response to the incident SW beam at a frequency $f=45$ GHz on the edge mode at a frequency $\nu =12.5$ GHz. The blue highlighted peaks correspond to the incident SW beam and its second harmonic. The red peaks represent the edge mode and its harmonics. SSP and CP are highlighted in green and purple, respectively. These frequencies are also marked with horizontal lines in (**c**). **(e)** Isofrequency construction illustrating the principle of selecting wavevectors of inelastically scattered SW beams on the edge mode propagating to the right, i.e., with $\kappa > 0$. The blue, green, and purple curves represent isofrequency contours for SWs at frequencies of the incident SW beam $f=45$ GHz, reduced in frequency by the edge mode frequency ($f-\nu$ or $f-\nu^\prime$) corresponding to SSP, and increased in frequency by the edge mode frequency ($f+\nu$ or $f+\nu^\prime$) corresponding to CP. The green and purple isofrequency contours are plotted for two edge mode frequencies, i.e., solid lines for $\nu =11$ and dashed lines for $\nu^\prime =15.5$ GHz. We keep the same color code as in the previous figures to represent the undergoing processes. The red, blue, green, and purple arrows represent successively the wavevectors of the edge SWs, the incident SW beam, and the inelastically scattered SW beams resulting from SSP and CP. In addition, we mark the group velocities of the SW beams with yellow vectors, which are normal to the curvatures of the contours. The angle between the group velocity and the normal to the interface of a given SW beam is denoted as $\Theta$ and it is the angle of beam propagation.

We analyze the dynamics of SWs in a 10 nm thick Py layer using micromagnetic simulations performed in the Mumax3 environment^57^. We use typical material parameters of Py ($\textrm{Ni}_\textrm{80}\textrm{Fe}_\textrm{20}$), namely $M_\textrm{S} = 800$ kA/m, $A_\mathrm {{ex}}=13$ pJ/m, but with reduced damping parameter $\alpha = 0.0001$ for easier analysis of SW propagation in the far field. The layer is semi-infinite, i.e., it has only one sharp edge and is nominally infinite in all other in-plane directions, Fig. 1(a). The external uniform magnetic field $B_0 = {\mu _0}H_0=300$ mT is applied perpendicular to the layer’s edge, ${\textbf {H}}_0 = H_0\widehat{{\textbf {y}}}$, which induces a demagnetizing field on the edge that locally lowers the effective static field, Fig. 1(b). This non-uniformity serves as a potential well in which the SW-localized mode can be confined^9,58,59^. Accordingly, the dispersion relation shown in Fig. 1(c) consists of a part representing a continuum of SWs freely propagating far from the interface and a distinct band representing the edge mode with frequencies downshifted with respect to the SW continuum. The gap between the modes is from 11 GHz to 15.5 GHz at wavenumber $k_x=0$. This implies that in this frequency range, the propagating SWs are confined to the system’s edge. This situation provides us with a straightforward way to excite only edge modes at low frequencies and to study the inelastic scattering of higher frequency SWs incident from the thin layer on the layer edge.

We study the inelastic scattering of a SW beam of frequency 45 GHz (corresponding to the wavelength of 35.7 nm), full width at half maximum 760 nm, incident obliquely at the edge where an edge mode of frequency $\nu$ is localized. Throughout the paper, the frequency of the SW edge mode is kept below the bottom of the SW continuum, 15.5 GHz, so the edge mode cannot leak the energy to the bulk of the Py layer. We place two antennas, which emit local oscillating magnetic fields to excite both types of SWs (see yellow stripes in Fig. 1a). The first antenna is placed at the very edge of the system and is responsible for exciting edge mode with frequency $\nu$. The second antenna is placed about 3.84 $\mathrm{\upmu m}$ from the edge and excites the SW beam with frequency $f=45$ GHz. The second antenna excites unidirectionally propagating SW beam towards the edge^60,61^ at a specific angle of incidence (angle between the wavevector of incident SW beam and the normal to the interface, i.e., the *y*-axis). More details are given in the “Methods” section.

In the numerical simulations, we change three parameters, the angle of SW beam incidence $\theta$ and edge mode frequency (which also changes the wavenumber of the edge mode $\kappa$), and the sign of $\kappa$. The angle of incidence is controlled by the orientation of the antenna relative to the edge of the layer. We use the following set of angles $\theta =\{30^\circ , 32.5^\circ , 35^\circ , 37.5^\circ , 40^\circ , 45^\circ \}$. We choose the sign of the edge mode wavevector by changing the position of the edge antenna with respect to the incident beam spot at the edge. If the edge antenna is placed to the left of the incident SW beam spot, the edge mode wavevector $\kappa$ is positive. If the antenna is to the right, $\kappa$ is negative. For each configuration with the chosen $\theta$ and sign of $\kappa$, we run a series of simulations with different edge mode frequencies. These frequencies are in the range of $\nu \in \langle 11, 15.5 \rangle$ GHz.

Figue 1d shows the SW spectrum of the system obtained in the steady state in the case of $f=45$ GHz, $\nu =12.5$ GHz, $\kappa > 0$, $\theta =30^{\circ }$. We display the maxima of the $m_z$ magnetization component at a given frequency, calculated with Fourier transform. Two peaks highlighted in blue correspond to the frequency of SW beam *f* and its second harmonics. Several peaks highlighted in red represent the edge mode $\nu$ and its higher harmonics^9^. Two peaks of the main interest are marked with green and purple colors. These correspond to the frequencies of SSP $f-\nu$ and CP $f+\nu$ respectively, which confirms the existence of these phenomena in the studied system. We focus on these waves throughout the paper. Apart from the mentioned peaks, we also see several other peaks at frequencies corresponding to higher-order nonlinear processes (e.g. $f-2\nu =20$ GHz). These are beyond the scope of this paper and will be omitted in further analysis.

**Figure 2:**
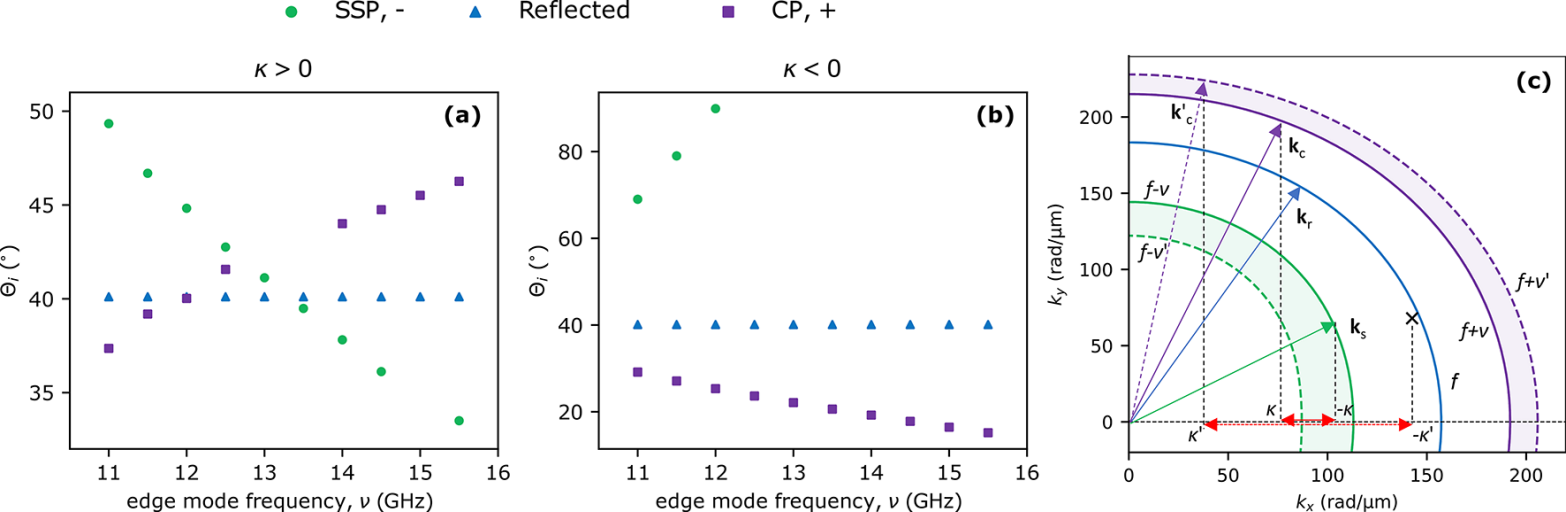
Dependencies showing the SW beams group velocity angles $\Theta _i$. **(a,b)** Angles of the group velocity of SW beams (angles of propagation) as a function of edge mode frequency $\nu$, blue triangles represent the reflected beam, green circles display SW beam created in SSP, and purple squares represent the SW beam created in CP. The results were obtained for the angle of incidence $\theta =30^{\circ }$ and for both, the edge modes with (**a**) $\kappa > 0$ and (**b**) $\kappa < 0$. **(c)** Isofrequency construction in the case of $\kappa < 0$. Here, for $-|\kappa ^\prime |$ there is no geometric solution in the SSP, which would correspond to SWs scattered on the higher frequency edge mode. Thus, for $\kappa < 0$ there is a critical value of $\kappa$ (and $\nu$) above which SSP is not allowed, as shown in (**b**).

For edge modes propagating to the right (i.e., $\kappa > 0$), the angles of the group velocity of the inelastically scattered beams incident at $\theta =30^{\circ }$ change monotonically with the change of $\nu$, see Fig. 2a (green circles and purple squares). Later in the text, we call the angle of the group velocity $\Theta _i$ as the angle of propagation. The angle of propagation of the beam generated in SSP (green circles) decreases monotonically with increasing $\nu$, and changes by $17^{\circ }$ in the examined range of $\nu$. The changes for the CP beams (purple squares) are opposite to SSP. Here the angle increases with increasing $\nu$, and in the investigated range of $\nu$ the angle changes by $10^{\circ }$. The results for other angles of propagation of the incident SW beams are qualitatively the same. These changes in the angle of the propagation of inelastically scattered beams can be explained using isofrequency contour analysis^15^. We present the considered inelastic scattering of SW beams on edge mode with $\kappa > 0$ on isofrequency plots in Fig. 1e. The blue curve represents all available solutions for the frequency *f* and $k_x>0$. The blue dashed arrow represents the wavevector associated with the incident SW beam (**k**_i_). The solid blue arrow marks the opposite quadrant of $(k_x, k_y)$ space and is plotted according to the rule of the conservation of wavevector tangential component ($k_x$), i.e., Snell’s law. It represents the wavevector associated with the reflected SW beam (**k**_r_), which must be located on the isofrequency contour for frequency *f*. The beam propagation directions are related to the directions of the group velocities $\textbf{v}_\textrm{g}$(*f*, **k**) (marked with orange arrows), which are perpendicular to the constant frequency contour for the corresponding wavevectors. Taking into account the conservation of energy ($f^\prime =f + \nu$ for CP and $f^\prime =f - \nu$ for SSP) and the conservation of the tangential component of the wavevector to the interface ($k_{\mathrm{c},x}=k_x + \kappa$ for CP and $k_{\mathrm{s},x}=k_x - \kappa$ for SSP), a similar construction can be made for inelastically scattered SWs. These contours, for SSP (green curves) and CP (purple curves), are marked for two examples of edge mode frequencies $\nu =11$ GHz and $\nu ^\prime =15.5$ GHz, solid and dashed lines, respectively. It can be seen that for the lower edge mode frequency, the propagation angle relative to the normal to the interface (*y*-axis) is smaller, which is consistent with the simulation results shown in Fig. 2a. A similar construction is done for SSP for the isofrequency contour $f-\nu$ and for the *x*-component of the wavevector equal to $k_{\mathrm{s},x}=k_x - \kappa$. Therefore, we observe that for the lower value of the edge SWs frequency, the angle of propagation of the inelastically scattered beam increases, which is also in agreement with Fig. 2a. Later in the text, we present dependencies of angles of propagation on the edge mode frequency for other simulation cases.

In the case of the left propagating edge mode, i.e. $\kappa < 0$, the SSP can only exist at frequencies $\nu$ below some critical value (see green dots in Fig. 2b where the waves formed in the SSP are only below 12 GHz), which depends on the angle of incidence. As shown in the isofrequency contour construction in Fig. 2c for $\theta =30^\circ,$ for $k_{\mathrm{s},x}=k_x-(-|\kappa |)$ corresponding to $f - \nu$ frequencies, there are solutions only for small $\kappa$ vectors. The angles of propagation of these rays are above $70^\circ$, i.e., their amplitude distribution overlaps with the interface, and therefore it is difficult to derive their trajectories. For the CP, the angle of propagation of the inelastically scattered beam decreases with increasing edge mode frequency (Fig. 2b) and varies by $14^{\circ }$ in the considered range of $\nu$. This is in agreement with the analysis of the changes in the group velocity directions shown in Fig. 2c. This situation is the opposite of the results obtained in the simulations with the positive value of the wavevector of the edge SW.

**Figure 3:**
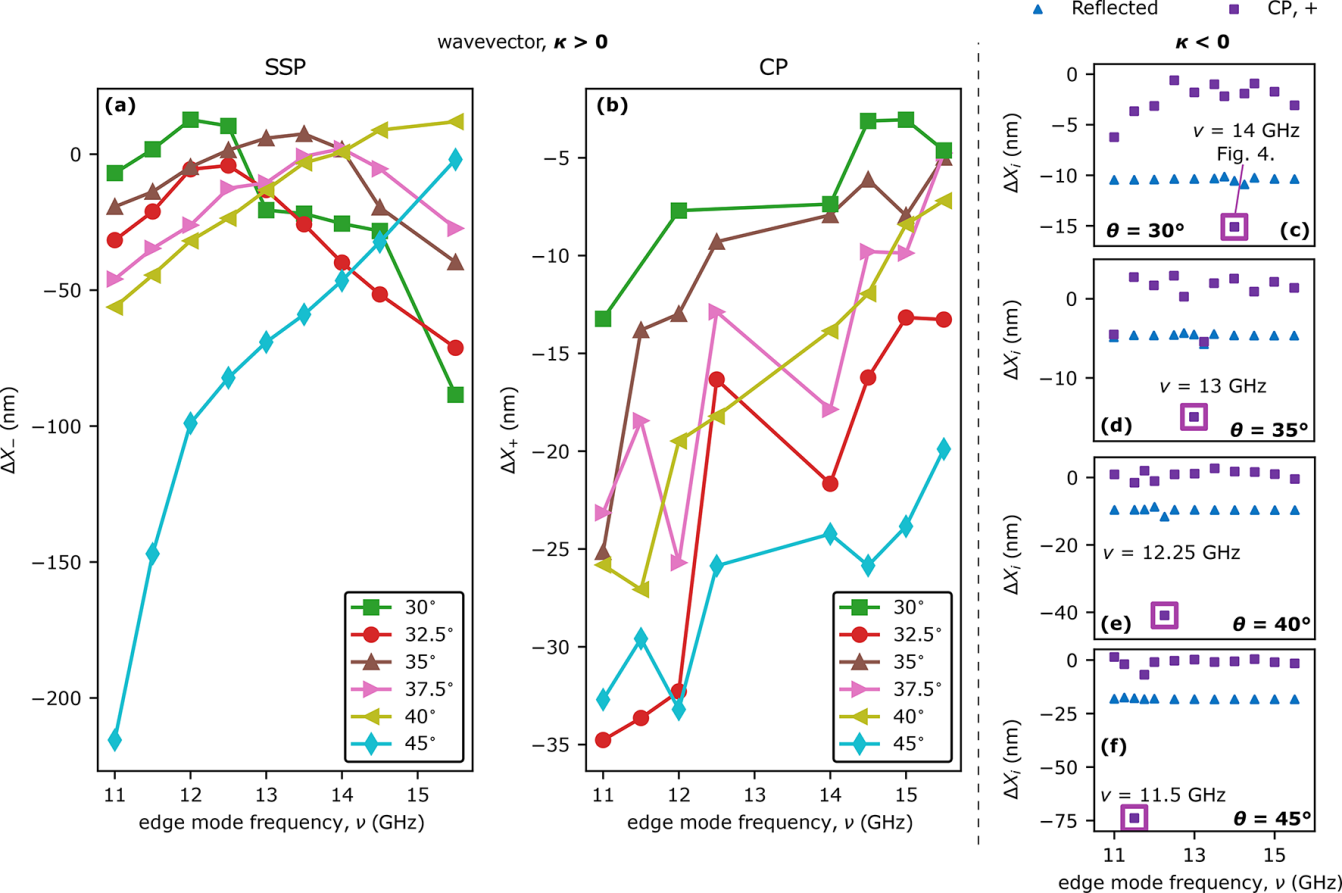
Dependencies of the lateral shifts $\Delta {X_{i}}$ along the interface of reflected and inelastically scattered beams for different angles of SW beam incidence ($\theta$), and edge SWs frequencies ($\nu$), and wavevector sign $\kappa >0$ and $\kappa <0$. On the left side of the black dashed line (subfigures (**a,b**)), lateral shifts of the scattered beams are presented in the case of edge mode with $\kappa > 0$. (**a**) Lateral shifts of the beams generated in SSP, and (**b**) in CP for all simulated angles of SW beam incidence. To the right of the black dashed line (subfigures (**c**)–(**f**)) the lateral shifts of the edge modes with $\kappa < 0$. The blue triangles show the lateral shifts of the elastically reflected beams and purple squares show the results corresponding to CP. The empty squares indicate the frequencies with the enhanced magnitude of $|\Delta X_+|$.

A detailed analysis of the rays of inelastically scattered SW beams shows that they are laterally shifted along the interface relative to the incident SW beam spot. This effect is analogous to the GH effect, which appears as a lateral shift of the elastically reflected wave beam with respect to the incident wave beam, e.g., electromagnetic waves^29–31^ or SWs^50,51,53^. The results of simulations with edge mode of wavevector $\kappa > 0$ and for $\kappa < 0$ for different incident beam angles are displayed in Fig. 3a,b and in c–f, respectively.

The value of the spatial shift for the elastically reflected SW beam, i.e., standard GH shift, weakly depends on the frequency of the edge mode besides one case described later in the paper. We report the smallest $\Delta {X}= -3.8$ nm for the angle of incidence $35^\circ$ and the biggest $-14.5$ nm at $45^\circ$ with $\kappa > 0$ (see, Fig. S1. in the Supplementary), for $\kappa > 0$ the GH shift variation is similar, i.e., between 4 nm (for $\theta =35^\circ$) and 15 nm ($\theta =45^\circ$) as shown in Fig. 3c–f. These are rather small values of the GH shift but close to the expectations. In a paper with conditions similar to those presented in this work, the scope of GH shift for SWs was reported to be up to 40 nm in a system SW wavelength of 60 nm^52^. Some other papers have reported significant values of GH-shift but for special cases, e.g., in strong magnetic fields and with incident SWs propagating at grazing angles to the interface^50,62^. However, there is no report so far on the GH shift of beams generated in inelastic scattering.

The scope of spatial shifts of the beams created in SSP scattered on the edge mode with $\kappa >0$ depends on the angle of the SW incidence beam and the edge mode frequency, see Fig. 3a (additional more detailed plots are also shown in Fig. S1. in the Supplementary). Interestingly, the function $\Delta {X}_{-}(\nu )$ is not monotonous. For instance, for $\theta \le 37.5^\circ$ the function $\Delta {X}_{-}(\nu )$ has in the analyzed $\nu$ range a maximum corresponding to a very small positive value of the GH shift with negative values (approx. $-20$ and -50 nm) at the sides of the considered range (11 and 15 GHz, respectively).

As the angle of incidence $\theta$ increases above $37.5^\circ$, the position of the extreme shifts towards the greater edge mode frequencies. Therefore, for angles $40^\circ$ and $45^\circ$, the $\Delta {X}_{-}$ dependence becomes monotonic in the analyzed edge mode frequency range. With decreasing $\theta$ from $37.5^\circ$, the maximum shifts to lower frequencies. Although the observed dependencies are regular, the reason for this behavior is unclear and difficult to explain without an appropriate analytical model.

Although the dependencies of $\Delta {X}_{-}(\nu )$ have maximum, the largest absolute magnitudes of the shifts $\Delta {X}_{-}$ are negative, and in the range of tens of nanometers. Thus, the spatial shifts are relatively small compared to the incident beam width. However, the wavelengths of the scattered beams generated in the SSP are in the range of 48 nm to 58 nm, varying slightly with different angles and edge mode frequencies. Thus, the reported spatial shifts are comparable to the scattered SW wavelengths. Exceptions to this rule are the results for $\theta =45^\circ$ and low edge mode frequencies which are in the range of hundreds of nanometers, e.g. for $\nu =11$ GHz $\Delta {X}_{-}=-215$ nm, which are several wavelengths of the scattered SWs. In the results presented in Fig. 3(a) we omit the edge mode frequency $\nu =15$ GHz as the frequency resulting in SSP is $f-\nu =30$ GHz, which is the second harmonic of edge mode excitation that overlaps with the bulk SW spectra thus interfering with the derivation of SW beam trajectory.

The dependencies of the spatial shifts of the beams generated in CP at $\kappa >0$ on $\nu$ are shown in Fig. 3b (additional more detailed plots are also shown in Fig. S1. in the Supplementary). For all angles of incidence $\theta,$ despite the variation in the lateral shift value $\Delta {X}_{+}(\nu )$, there is a general tendency for the magnitude of the negative $\Delta {X}_{+}(\nu )$ to decrease as the edge SW frequency increases. The scope of $\Delta {X}_{+}$ is smaller in comparison to the spatial shifts calculated for beams created in SSP, and are in the range from -35 nm to 0. These values are comparable or smaller in comparison to scattered SWs wavelengths, which are in the range of 28 nm and 30 nm for CP. In Fig. 3b, the results for frequencies $\nu =13$ GHz and $\nu =13.5$ GHz are not displayed because for these frequencies the amplitudes of the scattered beams are very low, and derivation of their trajectories is unreliable. The analysis of scattered SWs amplitude dependency on the edge mode frequency is beyond the scope of this paper and will be the subject of a further forthcoming study.

Figure 3c–f show the results of spatial shift of SW beams scattered on the edge mode with $\kappa <0$. In most of the cases, the SSP does not occur, as was explained before, and in the few cases where this process does occur the scattered beams are almost grazing the edge thus, the derivation of their spatial shifts is inaccurate. For that reason in the case of $\kappa <0$, we only present spatial shifts of beams created in CP. In all simulations with different angles of incidence, the derived spatial shifts of the scattered beams in CP are very small, in the range of a few nanometers, where the wavelengths of the scattered SWs are in the range of 26 nm to 29 nm. However, we can find a certain value of the edge mode frequency (14, 13, 12.25, and 11.5 GHz at $\theta =30^\circ$, $35^\circ$, $40^\circ$ and $45^\circ$, respectively) for which a significant enhancement of the GH shift value appears, see purple squares in Fig. 3c–f. The origin of this phenomenon will be explained in the following paragraphs.

**Figure 4:**
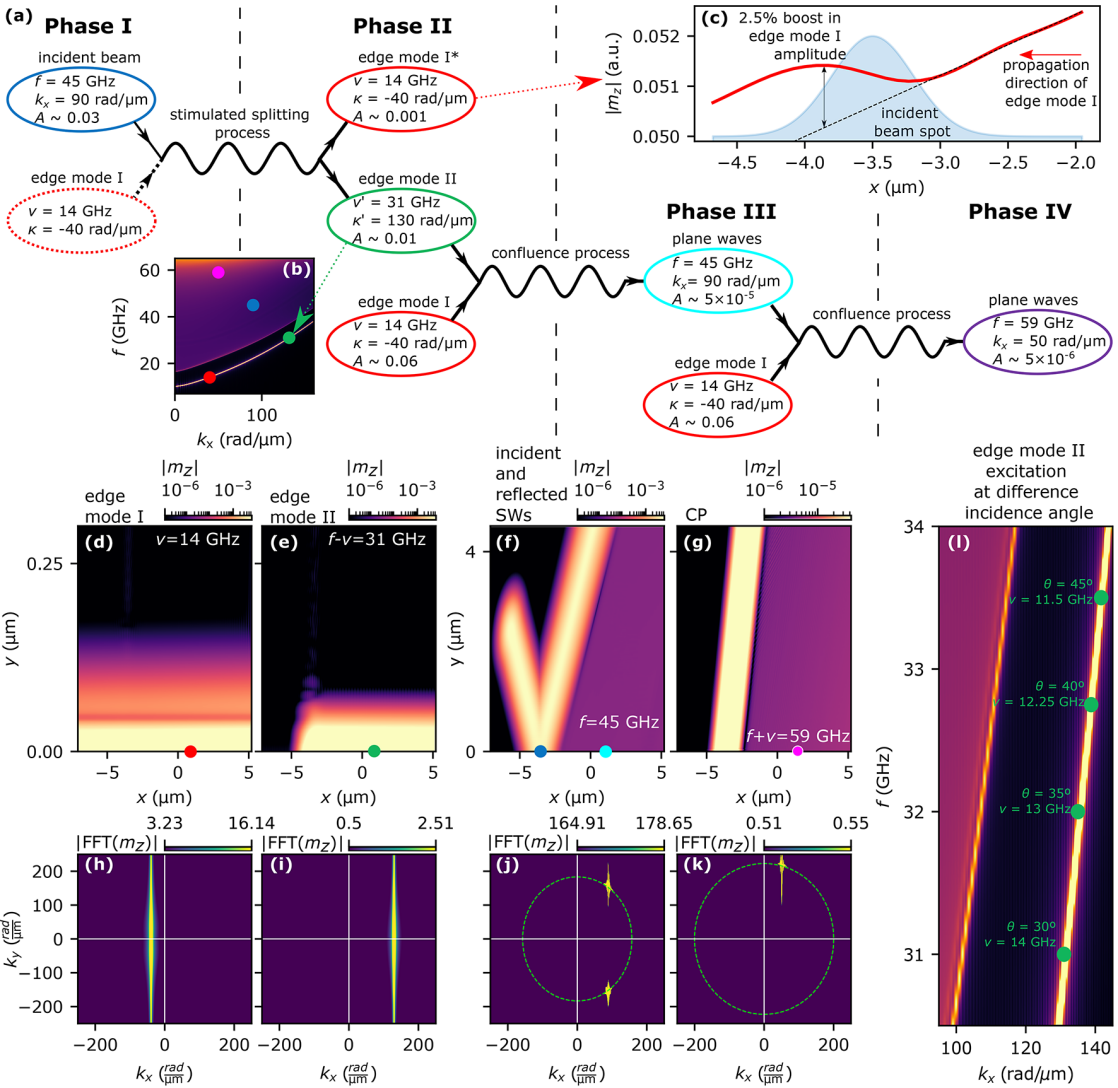
Cascade nonlinear excitation of SW leaky modes. (**a**) Scheme of nonlinear cascade process explaining the excitation of edge and plane SW in the system with an incident SW beam of frequency $f=45$ GHz propagating under angle $\theta =30^{\circ }$. The SW modes are symbolized with ovals whose colors indicate the modes of the same characteristics. The proposed cascade process is divided into four phases connected by SSP and two CPs. (**b**) Dispersion relation of the system with the marked modes shown in (**a**) (the color of the dots corresponds to the colors of the ovals). (**c**) Amplitude $|m_z|$ of the edge mode I (red, solid line) at the layer edge in the vicinity of the incidence spot of the incident SW beam. The black, dashed line is the extrapolation of the antenna-excited edge mode I behind the incident beam spot, i.e., for $x>-3~\mathrm{\upmu m}$. (**d–g**) Space distributions of $|m_z|$ at frequencies $\nu =14$ GHz, $f-\nu =31$ GHz, $f=45$ GHz, $f+\nu =59$ GHz, respectively. The dots indicate spots where SW modes amplitudes, *A* indicated in (**a**), were taken. (**h–k**) Results of the two-dimensional Fourier transform from the space to wavevector domain of SW amplitude distribution are shown in (**d–g**). Figures (**j,k**) additionally contain isofrequency contours that correspond to frequencies of SWs presented in these pictures. (**l**) Investigated system’s dispersion relation with marked frequencies for which excitation of SW plane waves occurs at different angles of incidence (this picture shares the colorbar with Fig.1b).

Let us first focus on the case shown in Fig. 3c, i.e., for the angle of incidence 30$^\circ$ and the frequency of the edge mode $\nu =14$ GHz. The spatial distributions of the SW amplitude at frequencies $\nu =14$ GHz, $f=45$ GHz, and $f+\nu =59$ GHz are shown in Fig. 4d, f, g, respectively. Surprisingly, in addition to the beams (incident and reflected at 45 GHz, and scattered at 59 GHz), we can also see a brightened region representing the plane waves with the same wavevector as the SWs from the beam. This effect can be explained as a result of a cascade nonlinear excitation of plane waves at the layer’s edge. Figure 4a shows a scheme of the proposed nonlinear cascade process in the case of an incident SW beam propagating at the angle $\theta =30^{\circ }$ and scattered on a propagating edge mode of frequency $\nu =14$ GHz. The first phase of this process is the SSP of the incident SW beam, marked by the blue oval, on the edge mode I, dashed red oval. As described before, there is no allowed solution to this process in the bulk of the Py layer. However, in this particular case, the result of the SSP is the generation of SWs with frequency $f-\nu =31$ GHz and wavevector $k_x-|-\kappa |=130$ rad/${\upmu }$m, which coincides with one of the allowed edge states in the system, as shown in Fig. 4b, later in this paper we will call this mode as the edge mode II. Figure 4e shows the spatial distribution of the SW amplitude of this mode, confirming its existence. According to the conservation of energy and momentum laws, this SSP must also generate SWs with frequency $\nu =14$ GHz and wavevector $|\kappa |=-40$ rad/${\upmu }$m corresponding to the primary excited edge mode (edge mode I, marked in red with an asterisk). The analysis of this edge mode I amplitude presented in Fig. 4c shows a 2.5% boost just behind the spot where the incident beam reaches the edge. It indicates the creation of new edge SWs that propagate along and oscillate in phase with the antenna-excited edge mode I, and can be considered as an amplification of the propagating edge mode^15^. In Fig. 4e, where edge mode II is presented, there is a distinctive gap left to the point of SW beam incidence ($x=-3.7$ ${\upmu }$m), which indicates that edge mode II is created at the SW beam incidence spot and propagates in opposite direction to edge mode I, Fig. 4d. It is evident by calculating the Fourier transform from the space to wavevector domain, as presented in Fig. 4h,i. Both of the edge modes have wavevectors of opposite signs and their numerical values agree with the analytically derived values shown in scheme Fig. 4a.

The excited edge mode II propagating along the edge interacts with the edge mode I excited directly by the antenna, Fig. 4a, it is Phase II. It causes CP to occur along the length of the edge beyond the point of SW beam incidence. The outcome of this process is the creation of a new SW at the edge with $f=45$ GHz and wavevector $k_x=90$ rad/${\upmu }$m, which corresponds to the incident SW beam. Taking into consideration the isofrequency contours, newly-created SWs at the edge also have to gain a wavevector component perpendicular to the edge, since there is no such solution for pure edge mode. For that reason, these SWs leak the energy from the edge and propagate into the bulk of the system with their wavefronts parallel to the reflected SW beam. It is shown in Fig. 4f, where an area of nonzero SW amplitude is right to the reflected beam. Additionally, the spatial Fourier transform of that distribution, shown in Fig. 4j, consists of only two distinctive peaks that overlap with isofrequency at $f=45$ GHz and correspond to the incident and reflected SWs. Thus, no SWs with new wavevectors at this frequency are excited in the system. Since we are dealing with a nonlinear cascade process in which the interaction of two edge modes of different frequencies ultimately leads to energy leakage from the edge, this process is a nonlinear analog of a leaky mode excitation, which we have already reported^61^. Therefore, we refer to this phenomenon as nonlinear cascade leaky mode excitation.

Furthermore, this newly created SW at the edge interacts with the antenna-excited edge mode I, Phase III. This interaction is yet another CP occurring at the system’s edge right to the incidence spot. In this CP new SW plane waves are excited, which propagate parallel to the scattered SW beam, Phase IV. We show this wave in Fig. 4g where the distribution of $|m_z|$ at frequency $f=59$ GHz has nonzero amplitude only to the right of the scattered SW beam created in CP, i.e., in the direction of propagating edge mode II. Fig. 4k presents the space-domain Fourier transform of the $|m_z|$ distribution. There is only one peak that coincides with the isofrequency contour of $f=59$ GHz confirming that the new SW plane wave has the same wavevector as the inelastically scattered SW beam.

In the scheme in Fig. 4a we wrote down the amplitudes (*A*) of all SWs types considered in the described cascade process. It points out that the amplitudes of created SWs in each phase are proportional to the product of the amplitudes in the previous phase. In Figs. 4d-g we mark positions where the $|m_z|$ amplitude values have been taken from the simulation results with circles, which colors correspond to the SWs modes shown in the Fig. 4a. The comparison between the incident beam and plane waves created in CP amplitudes of the plane waves created in CP shows a decrease in the range of $10^{4}$ factor over the course of three process phases. Despite such a minuscule magnitude of new SWs they seem to have a noticeable impact on the lateral shift of scattered beams as shown in Fig. 3c-f. Indeed, a closer analysis of Fig. 4f,g shows that there is a narrow, yet distinctive drop in SW amplitude between the beams and plane waves. Such a drop in amplitude is a result of the destructive interference of these two kinds of SWs, thus the newly excited SWs at the edge have to be significantly shifted in phase.

We also confirmed numerically that the nonzero amplitude background behind the scattered SW beams does not cause a false illusion of the beam shift. To confirm that adding a small-amplitude background to only one side of the Gaussian curve does not contribute the most to the calculated beam shift, we set up a numerical test. Namely, we added to a Gauss curve (with dimensions corresponding to the simulation results) a Heaviside step function with a height smaller by two orders of magnitude in comparison to the maximal amplitude of the Gauss curve. Then we run the same post-processing for this data as for simulated scattered beams. In the case of a numerically plotted Gauss curve, the addition of amplitude background alters the derived lateral shift by only a few nanometers. In the simulations, the scattered beams undergo lateral shifts up to tens of nanometers. These calculations confirm that adding a small-amplitude background does not change substantially the obtained values of lateral shifts of simulated SW beams.

In Fig. 4l we show a part of the system’s dispersion relation where the parameters of edge modes II are marked for all simulated angles of SW beam incidence. It is evident that in the investigated cases of SW beams scattered on the edge mode I with $\kappa <0$ only a limited number of $(\nu ,\kappa )$ and $(f,k_x)$ combinations will yield excitation of new edge modes. The reason for this is the relatively small width of the edge mode band in dispersion relation which allows only a narrow range of SWs to be excited at the edge. For that reason the enhancement of the GH shifts in Fig.3c-f exists only at a narrow range of edge mode I frequencies.

## Conclusions

In summary, we showed that the inelastically scattered SW beams on the edge wave confined to the edge of the ferromagnetic film undergo spatial shifts $\Delta X_{-,+}$ along the film edge, which we interpret as an analog of the GH effect. The obtained GH-like shifts are negative in the majority of cases, but dependencies of the $\Delta X_{-,+}(\nu )$ for the beams formed in SSP and CP processes are different. For beams generated in SSP and positive wavevector of the edge wave, the $\Delta X_{-}(\nu )$ dependence is parabolic like, with $\Delta X_{-} \approx 0$ at maximum and decreasing values for larger and smaller $\nu$. We found that for high-incidence angles, e.g., $45^\circ$, and edge modes of low-frequency with positive wavevectors, the spatial shifts of the beams created in SSP can even reach 200 nm, i.e., several wavelengths of the scattered SWs. For the beams generated in CP, the GH-like shift is small and there is a weak dependence of $\Delta X_{+}$ on $\nu$. However, we found a peculiar phenomenon at certain edge mode frequencies when the wavevector $\kappa <0$. In these cases, we observed an enhancement of the GH shift from a few nm to even tens of nm. Interestingly, this effect is associated with the excitation of new higher-frequency edge modes in a cascading nonlinear process, consisting of two additional CPs. In these CPs, there are generated SWs with frequencies and wavevectors that overlap with the band of the propagating SWs in the film, i.e., they radiate from the film edge and propagate as plane waves. Thus, the proposed process can be considered as a nonlinear version of magnonic leaky-mode excitation^61^. Even though the lateral shifts of scattered beams have small magnitudes and are not a significant factor in the physical realization of magnonic devices, they still serve as indirect indicators of other phenomena happening in the system. As presented in this paper, a visible increase of lateral shift of SW beams created in CP hinted an occurrence of the cascade process that, up-to-date, has not been described.

The results presented in this work contribute with nonlinear effects to a new subfield of magnonics called SW optic^63–67^. This paves the way for the development of optically-inspired magnonic logic devices^68,69^, especially for neuromorphic and edge computing components that require nonlinearity^2,3^, with high microwave frequency operation at the nanoscale, and, most importantly, low power consumption.

## Methods

### Micromagnetic simulations

In our research, we employ micromagnetic simulations performed in Mumax3 environment^57^ to solve the Landau-Lifshitz equation in the time domain. The system is modeled as a cuboidal layer of Py with dimensions $5.12\:{\upmu \textrm{m}} \times Y\:{\upmu \textrm{m}} \times 10\:{\upmu \textrm{m}}$ (along *x*, *y*, *z* axes respectively). The value *Y* varies in simulations with different incident SW beam’s angle of propagation, namely $Y =\{10.24\:{\upmu \textrm{m}},\:11.33\:{\upmu \textrm{m}},\:12.42\:{\upmu \textrm{m}},\:13.65\:{\upmu \textrm{m}},\:14.88\:{\upmu \textrm{m}},\:17.73\:{\upmu \textrm{m}}$ for angles $\theta =\{30^\circ , 32.5^\circ , 35^\circ , 37.5^\circ , 40^\circ , 45^\circ \}$ respectively. The material parameters of Py used in the simulations are $\alpha = 0.0001$, $M_\textrm{S} = 800$ kA/m, $A_\textrm{ex}=13$ pJ/m, which yield the exchange length of $\lambda _\textrm{ex}=5.69$ nm. We use the discretization grid $5~\textrm{nm} \times 5~\textrm{nm} \times 10~\textrm{nm}$ (along *x*, *y*, *z* axes respectively), which is shorter than $\lambda _\textrm{ex}$ in the in-plane coordinates of the layer. We place the Py layer in a uniform external magnetic field $B_0=300$ mT directed in-plane, opposite to the direction of the *y* axis. To simulate the infinitely long system along both directions of the *x* axis and positive direction of the *y* axis we increase the value of the damping parameter $\alpha$ up to value 0.5 parabolically over the width of 600 nm to prevent reflections at these edges.

The dispersion relation presented in Fig. 1c was obtained as described in Ref. ^9^. A small, two-dimensional antenna was placed at the edge of the system. The antenna introduces a locally oscillating external magnetic field with time and space distribution described by *sinc* functions with cut-off parameters $f_\textrm{cut}=100$ GHz and $k_\textrm{cut}=300$ rad/$\upmu$m. This magnetic field excites omnidirectional SWs in the system thus, the SWs propagate both in the bulk of the system and in the demagnetizing magnetic field dip at the edge. For the dispersion relation calculation, 1000 snapshots of the magnetization configuration were saved with a time step of $0.5/(1.1f_\textrm{cut})$.

To excite the SWs in the scattering simulations we use two antennas, one placed in the bulk of the Py layer and the second at the system’s edge. The first antenna has a rectangular-like shape and is rotated by an angle $\theta$ thus can be regarded in terms of $\left( x', y' \right)$ coordinates. It creates the incident SW beam aimed at the edge-localized mode. We used a mathematical formula to create this antenna in the simulations inspired by Ref. ^60^

1 $$\begin{aligned} \begin{aligned} B_{\textrm{ext}, y}(t,x',y') = A_\textrm{ant}(1-e^{-0.2 \pi f t})R(y')G(x') \\ \times [ \textrm{sin}(k y^{\prime})\textrm{sin}(2 \pi f t) + \textrm{cos}(k y^{\prime})\textrm{cos}(2 \pi f t) ], \end{aligned} \end{aligned}$$

where $A_\textrm{ant}=0.01B_0$ is the amplitude of the dynamic field, $R(y')=H(-y'+\frac{w_a}{2})H(y'+\frac{w_a}{2})$ is a rectangle function, which describes antenna’s shape along its $y^\prime$ coordinate ($H(y)$ is Heaviside step function, antenna’s width $w_a=360$ nm), $G(x')=\textrm{exp}(-\frac{x'^2}{4\sigma _x^{2}})$ is a Gaussian function defining antenna’s shape along the $x'$-axis ($\sigma _x=320$ nm), *k* is the wavevector and *f* is the frequency of the excited SWs. Such a formula creates a unidirectional SW beam of Gaussian envelope with FWHM$=760$ nm. The incident SW beam in all simulations has frequency $f=45$ GHz and the corresponding wavevector for this SW beam is derived from Kalinikos-Slavin formula for SW dispersion relation^70^ for each incident SW beam’s angle of propagation. We control the angle of the incident SW beam propagation (angle of the wavevector) by changing the rotation of the antenna, $\theta$, with respect to the edge of the system. In these investigations, we limit ourselves to the range of $\theta \in \langle 30^\circ , 45^\circ \rangle$. However, the calculated angles of propagation of the incident and reflected SW beams, $\Theta _i$ are different because of the SW anisotropy of propagation. This means that beams’ wavevectors and group velocities are not parallel. The second antenna is defined as a point source of Gaussian shape ($\sigma _\textrm{edge}=15$ nm) placed at the very edge of the system. The purpose of this antenna is to excite the localized edge modes characterized by frequencies in range $\nu \in \langle 11, 15.5 \rangle$ GHz. Depending on the desired wavevector of the edge mode $\kappa$ we place this antenna either below the point where the incident SW beam reaches the edge (positive $\kappa$) or above this point (negative $\kappa$).

The numerical simulations consist of three phases. In the first, we obtain a stable static magnetic configuration at the defined external magnetic field by minimizing the energy of the initial magnetic state. In the next phase, we run the dynamic part of the simulation when both antennas are turned on until the system reaches a steady state. The simulation time varies with the system’s length along its *y*-axis, $t =\{240~\textrm{ns}$, $270~\textrm{ns}$, $300~\textrm{ns},$ $325~\textrm{ns}$, $350~\textrm{ns},$ $420~\textrm{ns}\}$ for the incident SW beam angles $\theta =\{30^\circ ,32.5^\circ , 35^\circ , 37.5^\circ , 40^\circ , 45^\circ \}$ respectively. Finally, we save 800 snapshots of the magnetic configuration with ${dt}=5$ ps time step. We have also performed simulations of the described system with finer discretization through layer thickness to investigate the convergence of our results. For this purpose, we have used the following discretizations $5~\textrm{nm} \times 5~\textrm{nm} \times 5~\textrm{nm}$ and $5~\textrm{nm}~\times 5 \textrm{nm}~\times 1~\textrm{nm}.$ The obtained results differ only slightly (for almost all the cases the difference in lateral shifts are below 1 nm) in the numerical values but dependencies presented in the paper remain qualitatively the same.

### Data postprocessing

To process the data obtained in the micromagnetic simulations we use a self-developed code. We start with the spectral analysis of the scattered SWs by calculating the Fourier transform in the time domain using saved magnetic configuration snapshots for each simulation case. From this analysis, we obtain an insight into the frequencies of the processes that undergo in our system and the distribution of complex SW amplitude at a given frequency ($m_z(f)\in {\textbf {C}}$), therefore, allowing us to analyze both the amplitude and phase of SWs in each point of the simulated area. The time sampling was chosen to provide such resolution in the frequency domain to analyze the system’s response in all anticipated frequencies of $f\pm n\nu$, $n \in {\textbf {N}}^{+}$ configurations, namely $\delta t=5$ ps time step. In our calculations, we investigate the following frequencies $f=45$ GHz corresponding to the incident and reflected SW beams, $f-\nu$ corresponding to scattered beam in SSP, and $f+\nu$ corresponding to scattered beam in CP. Even though higher-order nonlinear processes are also present in the spectral analysis we omit them as they are beyond the scope of this paper.

**Figure 5:**
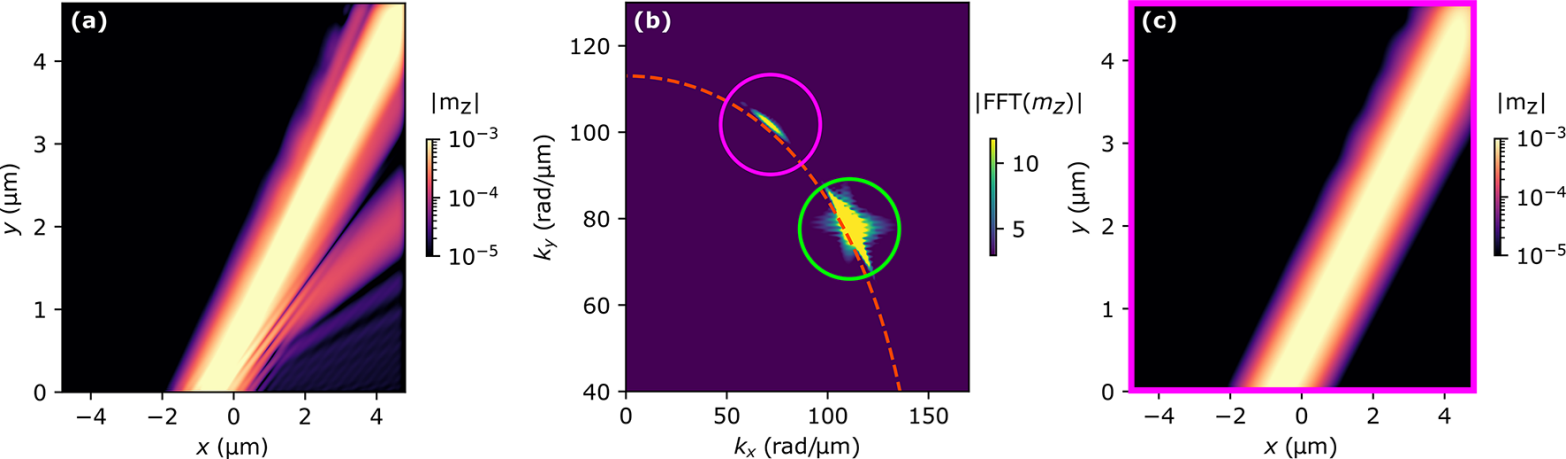
Numerical wavevector filter. (**a**) The $|m_z|$ amplitude space distribution of scattered SWs in SSP for $\theta =30^{\circ }$, $\kappa > 0$ and $\nu = 11$ GHz case with visible waves that are the result of secondary scattering on reflected edge mode. (**b**) Fourier transform of the scattered waves presented in (**a**). Two distinctive peaks are evident, one marked with a purple circle corresponds to SSP and the second, a green circle, corresponds to secondary scattering. (**c**) The simulation results after applying the wavevector filtering where only contribution from the wavevector of SSP is taken into consideration (purple circle in (b)).

The simulation results for $\kappa > 0$ and low edge mode frequency $\nu =11$ GHz proved to be problematic in further postprocessing. The reason for that was the unexpected reflection of low-frequency edge mode from the absorbing boundary conditions, where up to $10\%$ of edge mode amplitude was reflected. It caused a secondary scattering in the system and the creation of an additional SW beam of frequency corresponding to SSP but propagating under a different angle, as shown in Fig. 5a $|m_z|$ amplitude for SSP in $\theta =30^{\circ }$, $\kappa > 0$ and $\nu = 11$ GHz case. This new beam is a result of a CP with negative values of edge mode frequency and wavevector, as the reflected edge mode propagates in the opposite direction to the excited edge mode, thus resembling SSP in frequency but differing in wavevector of the scattered beam. To limit the effect of secondary scattering in our analysis we use an additional numerical filter that cuts off the contribution of SWs with different wavevectors than predicted by the investigated inelastic scattering processes. For that, we only take the contribution from the peak and its vicinity in spectral analysis that corresponds to the investigated process, as shown with the purple circle in Fig. 5b. To extract only the contribution for the desired peak and avoid numerical errors in the further steps of postprocessing we multiply the amplitude distribution by an amplitude mask in the shape of a two-dimensional Gaussian curve centered over the peak with a small spread. Then the inverse Fourier transform of the product of amplitude distribution and mask yields the SW amplitude space distribution without any undesired distortions in the system, as shown in Fig. 5c. This procedure was applied to the simulation results with low edge mode frequency, $\nu =11$ GHz to increase the precision of derivation of beams’ trajectories.

### Derivation of SW beams trajectories

To derive the trajectories of the investigated SW beams we use distributions of SW intensity in space for a given frequency and wavevector corresponding to incidence, reflection, SSP, and CP. We calculate all of the beams’ trajectories in the far field, in terms of geometry used in the simulations, $2~\mathrm{\upmu m}$ away from the edge. We fit the Gaussian function to the cross-sections of the SW $|m_z|$ intensity distributions at fixed *y*-coordinate. We used 200 cross-sections with an interval 5 nm, as the discretization grid used in the simulations. After fitting the Gaussian function to each cutline we save the position of the curve’s center of all the beams. Later, we use these coordinates to perform linear regression to fit line functions to them that we interpret as beams’ trajectories. In all simulation cases, the uncertainty of beam trajectory derivation depends on the spread of the beams’ center positions. In the simulation, we obtained well-collimated SW beams thus the uncertainty trajectory derivation is within 0.01 nm in all of the simulation cases. The derived trajectories are extended from the far field to the system’s edge where the position of the beams’ interception with the edge is determined. To calculate the spatial shifts of the scattered beams we take as a point of reference the position where the incident beam reaches the system’s edge. Thus the negative value of spatial shifts means that the given beam has its origin left to the incidence spot and the positive value means that the beam shifts to the right of the incidence spot. As the linear regression method calculates linear function fit in the continuous space, the final values of calculated space shifts of SW beams are not constrained by the discretization used in the simulations.

## Supplementary Information

Supplementary Information.

## Data Availability

Correspondence and requests for the data regarding this paper should be addressed to Krzysztof Sobucki (email address krzsob@amu.edu.pl). An example of simulation script in Mumax3 can be found in Zenodo repository, K. Sobucki, 2024, Zenodo, 10.5281/zenodo.13384164 (access via link https://zenodo.org/records/13384165).
