# Supplementary material for: Goos–Hänchen shift of inelastically scattered spin-wave beams and cascade nonlinear magnon processes
Source: Sci Rep. 2025 Feb 14;15:5538. doi: 10.1038/s41598-025-86879-y (PMC11829016; doi:10.1038/s41598-025-86879-y)
Supplement: Supplementary file 1 — Supplementary Information. [file 41598_2025_86879_MOESM1_ESM.pdf]

Goos-Hänchen shift of inelastically scattered spin-wave beams and cascade nonlinear magnon processes - Supplementary Material

Krzysztof Sobucki[1,*], Igor Lyubchanskii[**], Maciej Krawczyk[1], and Paweł Gruszecki[1]

[1]Institute of Spintronics and Quantum Information, Faculty of Physics, Adam Mickiewicz University, Uniwersytetu Poznańskiego 2, 61-614 Poznań, Poland
[*]krzsob@amu.edu.pl
[**]in association with Adam Mickiewicz University

Supplementary Material

More detailed the dependence of the propagation angle Θ_i (angle of the group velocity relative to the normal to the edge) of the reflected beam and the beams resulting from CP and SSP processes on the angle of incidence θ as well as on the edge SW frequency ν, see upper panels of each subplot in Fig.S1. Similar dependencies for GH shifts for both reflected and inelastically scattered beams in CP and SSP can be seen in the lower panels of each subplot in Fig.S1. This is a more detailed representation of the results shown in Figs. 3.(a,b). There we present the angles of scattered SW beam propagation and their lateral shifts at the interface in paired sub-figures for each SW beam incidence angle θ. We keep the same color code in all sub-figures, where blue triangles correspond to reflected SW beams and green circles and violet squares correspond to scattered SW beams generated in SSP and CP.

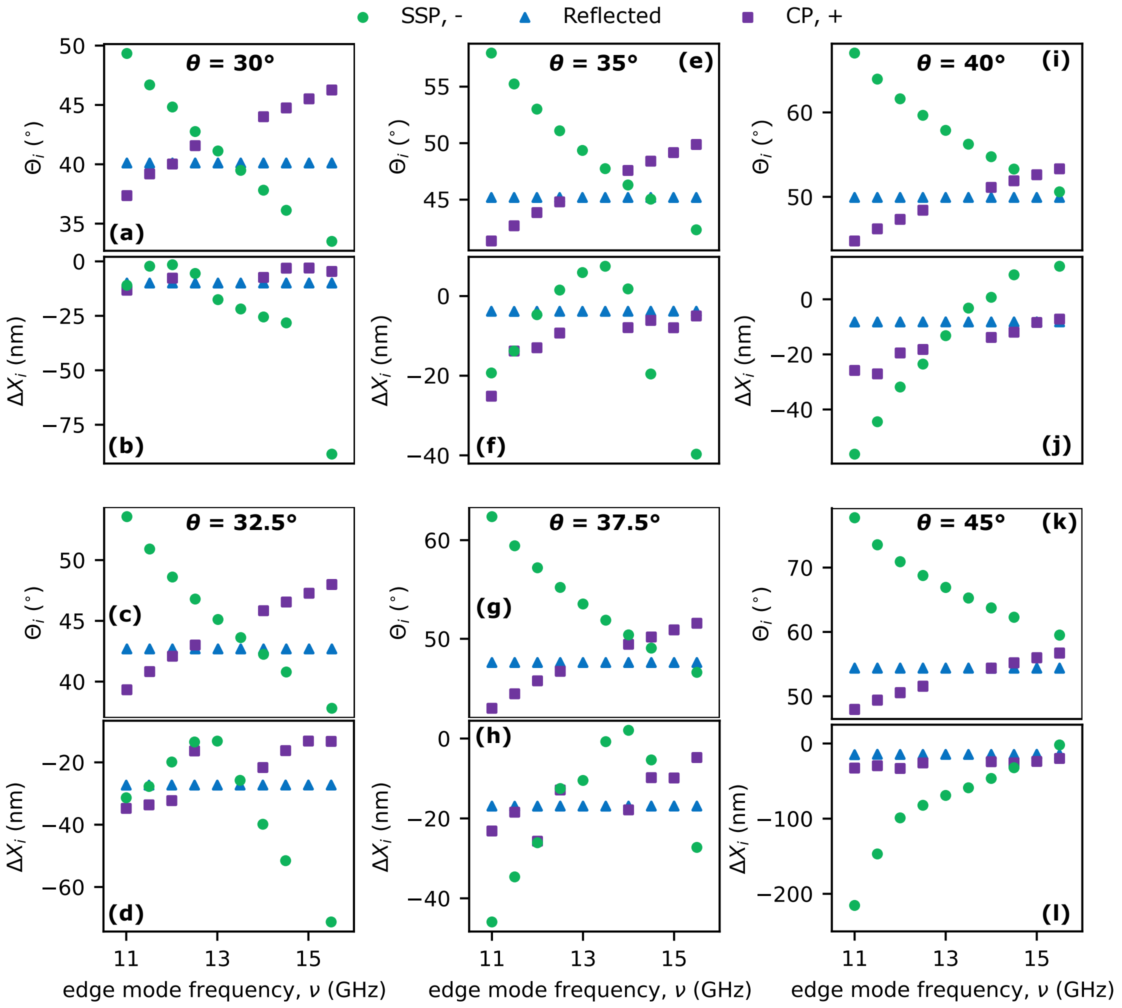

Figure S1. Dependencies of the propagation angle Θ_i (top panels) and a beam displacement along the interface (bottom panels) as a function of the edge SW frequency ν for the edge SWs propagating to the right ($\kappa > 0$), and for several different angles of incidence θ. The blue triangles display the results for reflected SW beams. The green circles and violet squares show the results of scattered SW beams generated in SSP and CP, respectively. The subfigures representing both the angles of beam propagation and their lateral shifts at the edge are paired according to the angle of SW beam incidence θ.
